# *Arabidopsis GLASSY HAIR* genes promote trichome papillae development

**DOI:** 10.1093/jxb/ert287

**Published:** 2013-09-07

**Authors:** Bangxia Suo, Stephanie Seifert, Viktor Kirik

**Affiliations:** School of Biological Sciences, Illinois State University, Normal, IL 61790, USA

**Keywords:** Cell wall, light reflection, papillae, trichomes.

## Abstract

Specialized plant cells form cell walls with distinct composition and properties pertinent to their function. Leaf trichomes in *Arabidopsis* form thick cell walls that support the upright growth of these large cells and, curiously, have strong light-reflective properties. To understand the process of trichome cell-wall maturation and the molecular origins of this optical property, mutants affected in trichome light reflection were isolated and characterized. It was found that *GLASSY HAIR* (*GLH*) genes are required for the formation of surface papillae structures at late stages of trichome development. Trichomes in these mutants appeared transparent due to unobstructed light transmission. Genetic analysis of the isolated mutants revealed seven different gene loci. Two—*TRICHOME BIREFRINGENCE* (*TBR*) and *NOK* (*Noeck*)*—*have been reported previously to have the glassy trichome mutant phenotype. The other five *glh* mutants were analysed for cell-wall-related phenotypes. A significant reduction was found in cellulose content in *glh2* and *glh4* mutant trichomes. In addition to the glassy trichome phenotype, the *glh6* mutants showed defects in leaf cuticular wax, and *glh6* was found to represent a new allele of the *eceriferum 10* (*cer10*) mutation. Trichomes of the *glh1* and *glh3* mutants did not show any other phenotypes beside reduced papillae formation. These data suggest that the *GLH1* and *GLH3* genes may have specific functions in trichome papillae formation, whereas *GLH2*, *GLH4*, and *GLH6* genes are also involved in deposition of other cell-wall components.

## Introduction

Plant cell walls perform a variety of essential functions, including mechanical support of individual cells and plant organs, protection against pathogens and abiotic environmental damage, and control of anisotropic cell expansion. These functions depend on physical and chemical properties of cell-wall components that provide strength and plasticity, form a protective surface, and recognize and transduce signals (Carpita and Gibeaut, 1993; Braam, 1999; Jones and Takemoto, 2004; Vorwerk *et al.*, 2004; Ellis *et al.*, 2010). It is estimated that about 10% of plant genes are devoted to synthesis, remodelling, or turnover of cell-wall components (McCann and Rose, 2010).

Although many genes involved in cell-wall metabolism have been identified, there are still many gaps in our understanding of cell-wall formation, including coordination of different biochemical activities, temporal and spatial regulation of biosynthetic processes, targeted deposition of wall components, and three-dimensional organization. Also the generic model of cell-wall organization and composition is not universal, and cell walls are strongly divergent depending on the type of differentiated cells (Keegstra, 2010). Analysis of cell-wall formation in different cell types is important to uncover developmental control mechanisms of cell-wall formation and to reveal molecular processes underlying natural variation in cell-wall organization.

*Arabidopsis* trichomes are specialized leaf epidermal cells that grow up to 0.5mm in height and form a cell wall of an impressive 1 μm thickness (Marks *et al.*, 2008). Large trichome size, stereotypic cell shape, accessibility for macroscopic observations, and dispensability for plant growth have facilitated the identification and functional characterization of >40 genes with loss-of function trichome phenotypes (Marks *et al.*, 2009). Genetic screens for trichome phenotypes have been instrumental in identification of genes involved in trichome development, as well as genes with essential functions in plant growth and development.

*Arabidopsis* trichomes provide an excellent model system to study the cell wall. Histochemical staining, transmission electron microscopy, and monosaccharide analyses have indicated that trichome cell walls are rich in pectin and cellulose, contain lignin and mannose-containing polysaccharides, and are covered with cuticular wax (Marks *et al.*, 2008). Transcriptome analysis studies have shown that genes involved in cell-wall function, biosynthesis, and structure are expressed at high levels in trichomes (Jakoby *et al.*, 2008; Marks *et al.*, 2009).

One peculiar feature of a mature trichome cell wall is the presence of primary cell-wall characteristics, including high pectin content (Marks *et al.*, 2008) and activity of primary-wall *CesA* genes (Betancur *et al.*, 2010). Consequently, mutations in secondary-wall *CesA* genes had no effect on thickening or birefringence of trichome walls (Betancur *et al.*, 2010). On the other hand, similar to other cells, trichome secondary-cell-wall thickening involves the formation of crystalline cellulose microfibrils that can be detected with polarized light as light birefringence (Potikha and Delmer, 1995; Nishikawa *et al.*, 2008; Betancur *et al.*, 2010). This mixture of primary and secondary cell-wall features in *Arabidopsis* trichomes provides an opportunity to uncover essential mechanisms of primary cell-wall formation, as well as to analyse processes important for the secondary cell wall, such as cellulose organization, lignin formation, and cuticular wax deposition.

Another peculiarity of trichome cell walls is the formation of surface papillae. Papillae form as subcuticular depositions during the cell-wall maturation phase of trichome development (Hulskamp *et al.*, 1994; Szymanski *et al.*, 1998; Esch *et al.*, 2003; Marks *et al.*, 2009). Mutants with strong papillae defects, such as *trichome birefringence* (*tbr*), *noeck* (*nok*), *glabrous 3-shapeshifter* (*gl3-sst*), and *homeodomain glabrous 2* (*hdg2*) have been reported (Potikha and Delmer, 1995; Esch *et al.*, 2003; Jakoby *et al.*, 2008; Marks *et al.*, 2009). Cloning of the corresponding genes, however, has not shed light on the mechanisms of papillae formation, composition, or function.

Trichomes of *tbr*, *nok*, *gl3-sst*, and *hdg2* mutants appear more transparent or ‘glassy’ when viewed in white light. This phenotype has also been reported for mutants *chablis* (*cha*), *chardonnay* (*cdo*), and *retsina* (*rts*) (Hulskamp *et al.*, 1994), and for mutants with stunted trichomes, such as *glabrous2* (Marks *et al.*, 2009), *underdeveloped trichomes1* (*udt1*) (Haughn and Somerville, 1988), *cpr5* (Brininstool *et al.*, 2008) and *midget*/*bin4* (Kirik *et al.*, 2007). Trichomes of *tbr* and *cpr5* mutants were shown to have reduced birefringence, indicating defects in paracrystalline cellulose formation.

The glassy trichome phenotype was used here to identity genes important for trichome cell-wall maturation. Five new *glassy hair* (*glh*) mutants were isolated. All of the *glh* mutants showed defects in papillae formation. Trichomes of *glh* mutants transmitted more light, suggesting papillae function in light scattering. Trichomes in *glh1* and *glh3* mutants displayed cell-wall defects restricted to papillae formation. Trichomes in *glh2* and *glh4* mutants deposited less cellulose, and *glh6* mutants showed a cuticular wax deposition defect indicating that the *GLH2*, *GLH4*, and *GLH6* genes have more pleiotropic functions in cell-wall formation. Our mapping results suggest that *GLH* genes represent loci with uncharacterized functions in deposition of papillae cell-wall materials and crystalline cellulose formation in maturing trichomes.

## Material and methods

### Plant strains and growth conditions

All plants were grown at 22 °C in a growth chamber or in the greenhouse. Seedlings were grown aseptically on plates containing Murashige and Skoog (MS) medium. Columbia-0 (Col-0) was used as a wild-type ecotype in experiments. Landsberg erecta (Ler) ecotype was used for mapping crosses. Seeds of the *hdg2* mutant were obtained from David Marks (University of Minnesota, MN, USA), *cer10-2* seeds were obtained from Ljerka Kunst (University of British Columbia, BC, Canada), and *tbr* seeds were provided by the *Arabidopsis* Biological Resource Center (ABRC).

### Genetic characterization of mutants

Mutants with transparent leaf trichomes were isolated using ×10 magnification in a stereomicroscope. M2 sibling families collected from 2400 individually harvested ethyl methanesulfonate (EMS)-mutagenized Col-0 plants were screened. Mutants with glassy phenotypes were crossed with each other for complementation tests. To create a double mutant of *hgd2* and *glh3*, which have indistinguishable glassy phenotypes and similar papillae defects, we screened the F2 generation for plants with glassy phenotype that were hemizygous for a T-DNA insertion in the *HDG2* gene. Double mutants were selected in the F3 generation using PCR to identify segregating *hdg2* homozygous plants with primers flanking the T-DNA insertion in the *HDG2* gene: HDG2-LP2, 5′-CACCAAAGGCATGACCAGTA-3′, and HDG2-RP2, 5′-CTCAAACTATCCACTTATGTCTCCT-3′. The *hdg2 glh3* double-mutant genotype was verified by crossing with both parent mutant plants.

For genetic mapping, *glh* mutants were crossed with Ler plants. Plants with a glassy phenotype were selected in F2 populations. Genomic DNA was isolated from 20–50 F2 plants. The map positions of the glassy mutations were determined relative to simple sequence length polymorphisms and cleaved amplified polymorphic sequences (CAPS) markers from the the Arabidopsis Information Resource (TAIR) database (http://www.arabidopsis.org/servlets/Search?action=new_search&type=marker), or using newly designed CAPS and derived CAPS (dCAPS) markers (dCAPS finder 2.0 program; http://helix.wustl.edu/dcaps/dcaps.html) based on single-nucleotide polymorphisms between Col-0 and Ler found at the TAIR webpage (http://www.arabidopsis.org/).

### Trichome isolation

To ensure that plant material was from the same developmental stage, fully expanded rosette leaves were collected from plants that had not bolted but already had flower buds. Trichomes were isolated from these leaves using a previously described method (Marks *et al.*, 2008). After isolation, trichomes were mixed thoroughly in PBS buffer. Three replicates of 10 μl each were pipetted from the suspension and trichomes were counted to estimate cell density. An estimated 2000 trichomes were aliquotted for trichome cellulose testing experiments.

### Cellulose quantification

Relative cellulose amounts were quantified using the Updegraff method (Updegraff, 1969) as follows. Prior to collection of material, plants were placed in the dark for 60–72h to reduce starch content. One fully expanded leaf of 4-week-old plants was used for leaf cellulose quantification. Leaves were incubated in 70% ethanol at 98 °C, dried in acetone, and their weight measured. To remove soluble contents, leaves and isolated trichomes were placed in 300 μl of acetic-nitric reagent at 98 °C for 30min. The plant material was then washed in water followed by two acetone washes. For the trichome cellulose detection experiment, trichome numbers in each tube were determined. Dried plant material was treated with 30 μl of 67% H_2_SO_4_ and vortexed to dissolve the samples. Next, 150 μl of water was added and mixed thoroughly, and the samples were placed on ice. Anthrone reagent (Acros Organics) was prepared on the day of the experiment, and 300 μl was added to each tube, mixed, and heated at 98°C for exactly 5min, and then transferred back to ice to stop the reaction. For leaf cellulose testing, 100 μl of the above solution was diluted with 700 μl of 67% H_2_SO_4_. For trichome cellulose testing, 150 μl of the solution was diluted with 650 μl of 67% H_2_SO_4_. Absorbance at a wavelength of 620nm was measured using a spectrophotometer.

### Microscopy and histology

For trichome opacity determination, fully expanded leaves of 3-week-old plants were mounted in a small drop of water and covered with a cover slip. Images were taken from trichomes positioned on the leaf edge so that the light was transmitted only through the trichome and not though the leaf. Trichomes with stronger light reflection formed a darker image. ImageJ software was used to measure the light differential between trichomes and the background. All images that compared mutants with wild type were taken under identical light and camera settings.

Before observing isolated trichomes by polarized light microscopy, trichomes were pre-treated with an acetic-nitric reagent. This treatment removes cuticular waxes, as they form crystalline structures (Koornneef *et al.*, 1989; Haslam *et al.*, 2012) and can potentially interfere with birefringence from crystalline cellulose. Trichomes were placed on a microscope slide between the crossed polarizer and analyser. Samples were rotated relative to polarization filters to allow the maximum amount of light to be transmitted, and images were acquired with a CCD camera.

For quantitative analysis of trichome birefringence, mean pixel grey values for the middle part of trichome branches were measured and background values were subtracted.

For scanning electron microscopy trichome imaging, fully expanded leaves from 3-week-old plants were used. Images were taken under low-vacuum at 0.98 torr pressure, 12.5kV, and 3.0 spot size. Plant tissue was placed on the stage cooled to 2 °C. Magnification of ×200 was used for whole-trichome images, ×800 for papillae density, and ×250 for papillae coverage.

Toluidine blue (TB) staining of cell-wall polysaccharides was performed as described previously (Tanaka *et al.*, 2004). Plants were grown on MS plates for about 2 weeks, and 0.05% (w/v) solution of filtered (0.2 μm pore size) Toluidine Blue O (Sigma) aqueous solution was then poured into the plates until the plants were submerged. The TB solution was removed after 2min, and plates were washed with water to remove the residual TB.

In all statistical tests, a Mann–Whitney non-parametric two-tailed test was used to determine the significance of measured differences.

## Results

### Isolation of glassy hair mutants

To uncover genes involved in trichome cell-wall maturation, we screened EMS-mutagenized populations of *Arabidopsis* for plants with glassy-appearing trichomes of normal size and morphology. These selection criteria focused our screen on identification of genes with a function during the trichome maturation phase, as opposed to genes required for progression through earlier stages of trichome development. We isolated ten *glh* mutants from the 2400 M2 sibling families of individually harvested EMS-mutagenized Col-0 plants. Crossing into the Col-0 wild type revealed that all *glh* mutants segregated with a 1:3 (mutant:wild type) ratio in the F2 generation, consistent with recessive and monogenic mutations.

These mutants were divided into three groups based on their trichome shape. The first group of six mutants had glassy trichomes of wild-type size and shape. Complementation tests within this group showed that the mutants were affected in five different genes: *GLH1*, *GLH2*, *GLH3*, *GLH4*, and *GLH5*.

**Fig. 1.**
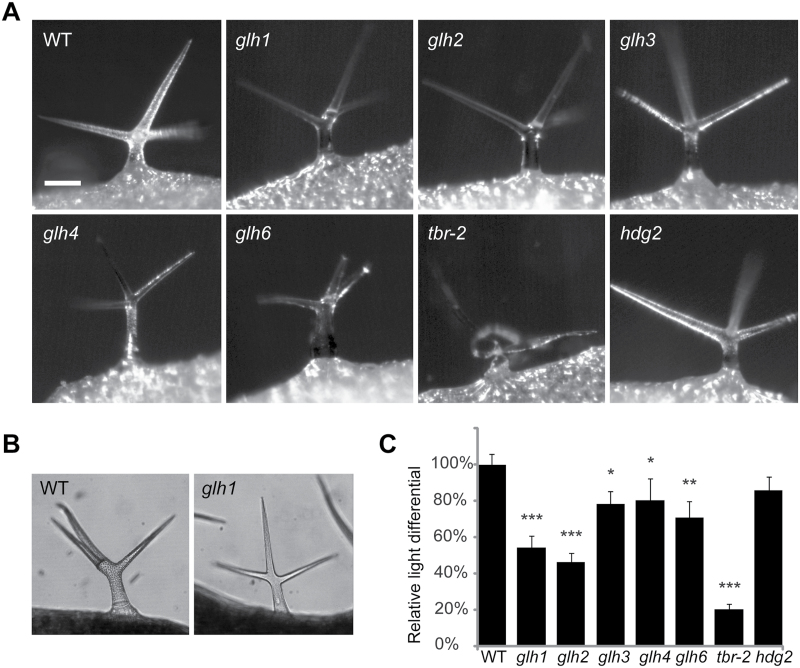
Glassy trichome phenotype of *glh* mutants. (A) Fully expanded leaves of 3-week-old plants were used to document trichome phenotypes. Trichomes of *glh1* and *glh2* mutants were the most transparent. The *hdg2* mutant (Marks *et al.*, 2009) had a weak glassy phenotype. Note a collapsed trichome in the *tbr-2* mutant. Bar, 100 μm. (B) Examples of representative images that were used to measure differentials between light transmitted through the trichome stem and the background. (C) Reduced trichome opacity of the *glh* trichomes. As a measure of trichome opacity, we calculated light intensity differential between light that passed through a trichome stem and the background. The lower opacity of *glh* mutants indicated higher transparency. The numbers of different trichomes used for measurements were: *n*=15 for wild type and *glh3*; *n*=13 for *glh1*, n=17 for *glh2* and *glh4*; *n*=19 for *glh6* and *hdg2*; and *n*=20 for *tbr-2*. All *glh* mutants and *tbr-2* showed significant differences in trichome opacity: ***P<0.001, for *glh1*, *glh2*, and *tbr-2*; **P<0.01 for *glh6*; and *P<0.05 for *glh3* and *glh4*. The decrease in *hdg2* opacity was not significant (*P*=0.4). Error bars show SEM. WT, wild type.

To test for genetic complementation with previously described glassy mutants with wild-type trichome shape and size, mutants of the first group were crossed with *tbr* (Potikha and Delmer, 1995) and with *hdg2* (Marks *et al.*, 2009) mutants. The *glh5* mutation did not complement the *tbr* mutation, suggesting that it represents a new allele of this mutant. Hence, we renamed *glh5* as *tbr-2*. Curiously, we found that mature trichomes collapsed in the *tbr-2* mutants (Fig. 1A), suggesting that trichome cell walls could not support the upright stature of these cells. We could also find occasional collapsing trichomes in the original *tbr* mutants, suggesting that *tbr-2* is a stronger loss-of-function allele of the *TBR* gene.

**Fig. 2.**
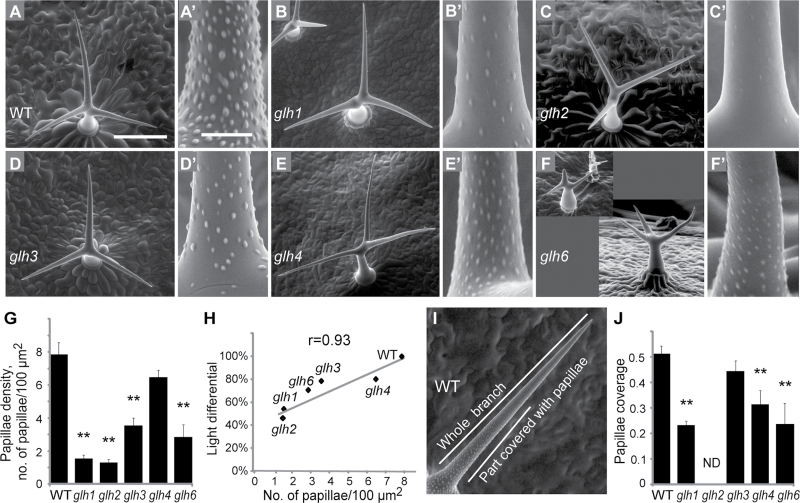
Papillae defects in *glh* mutants. (A–F) Scanning electron microscopy images of trichomes. (A′–F′) Higher-magnification images of trichome branches. (A′) Fully developed papillae in the wild type. (B′–F′) Papillae defects in *glh1*, *glh2*, *glh3*, *glh4*, and *glh6* mutants, respectively. (G) Papillae density determined as the number of papillae in 100 μm^2^ on trichomes imaged at ×800 magnification. The number of trichomes used in the measurements were: *n*=5 for WT, *glh1*, *glh3*, and *glh4* and *n*=15 for *glh6*. (H) Positive correlation between papillae density and trichome opacity measured as light differential in *glh* mutants and the wild type. Pearson’s correlation coefficient, *r*, was 0.93. (I) Determination of papillae coverage as the fraction of trichome branch length covered with papillae. The length of the trichome branch covered with papillae discernable at ×250 magnification was divided by the whole length of the trichome branch. (J) Papillae coverage in *ghl* mutant trichomes. No papillae were visible on trichomes of the *glh2* mutant at ×250 magnification. The numbers of trichomes used for measurement were: *n*=5 for WT, *glh1*, and *glh3*; *n*=8 for *glh4*; and *n*=15 for *glh6*. Bars, 200 μm (A–F); 20 μm (A′–F′). **P<0.01. Error bars in (G) and (J) show SEM. WT, wild type.

The second group contained three mutants that showed variable phenotypes, ranging from wild-type-like to small swollen trichomes with rounded tips (Fig. 1A and Fig. 2F). Genetic complementation tests revealed that all three mutants were affected in the same gene, which we named *GLH6*.

One glassy mutant had trichomes of wild-type size with increased branch number, resembling the *nok* mutant (Folkers *et al.*, 1997; Jakoby *et al.*, 2008). Genetic cross with the *nok-122* mutant allele did not show complementation, suggesting identification of a new *nok* allele, which we named *nok-2*.

Taken together, genetic analysis suggested that the *GLH1*, *GLH2*, *GLH3*, *GLH4*, and *GLH6* loci represent separate gene loci with unreported functions during trichome cell-wall maturation.

### The glassy trichome phenotype correlates with papillae development defects in *glh* mutants

We hypothesized that the visually perceived glassiness of trichomes reflects increased transparency of the cells. To test this hypothesis we measured the intensity of light transmitted through wild-type and *glh* mutant trichomes.

Trichomes in wild-type plants appeared darker and we measured higher light differentials between trichomes and the background light, indicating higher opacity than in *glh* mutants (Fig. 1A–C). The lowest light differentials were found in *glh1*, *glh2*, and *tbr* mutant trichomes, correlating with the stronger glassy appearance of the mutants. Trichomes of *glh3*, *glh4*, and *glh6* mutants appeared less glassy under the stereomicroscope and were more opaque than *glh1* and *glh2* trichomes (Fig. 1A, C).

It has been hypothesized that a reduction in papillae causes the glassy trichome phenotype (Jakoby *et al.*, 2008). Papillae are numerous round-shaped bumps on trichome walls that can increase light scattering due to an uneven cell surface. In their absence, trichome walls will be smoother and scatter less light. Previous studies showed that *nok*, *gl3-sst*, and *hdg2* mutants with a glassy appearance of trichomes formed fewer trichome papillae (Esch *et al.*, 2003; Jakoby *et al.*, 2008; Marks *et al.*, 2009).

We found that all *glh* mutants were defective in papillae formation (Fig. 2A–F). To quantify the defects, we measured papillae density and coverage of trichome branches in *glh* mutants (Fig. 2G–J).

On average, *glh1* trichomes formed 1.6 papillae per 100 μm^2^ trichome area, and *glh2* trichomes formed 1.3 papillae. This corresponds to a 5 fold (for *glh1*) and a 6 fold (for *glh2*) reduction compared with wild type (7.85 papillae per 100 μm^2^). The less transparent *glh3* and *glh4* mutants formed 3.6 and 6.5 papillae 100 per μm^2^ corresponding to a 2.2- and a 1.3-fold reduction in density compared with the wild type.

The density of papillae correlated strongly with trichome opacity (Fig. 2H). The most transparent *glh1* and *glh2* trichomes showed the strongest defects in papillae development, while the weaker glassy mutants *glh3* and g*lh4* showed a moderate decrease in papillae density and coverage.

The papillae coverage of *glh1* trichome branches was decreased by 55% compared with that of the wild type. Underdeveloped papillae in *glh2* were not discernable in ×250 magnification scanning electron microscopy images, precluding us from measuring papillae coverage in the mutant. Papillae coverage was decreased by 13% in the *glh3* mutant and by 39% in the *glh4* mutant, and this difference was statistically significant for the *glh4* mutant (Fig. 2J). In addition to defects in papillae density and coverage, the *glh1*, *glh2*, *glh4*, *and glh6* mutants formed less-developed flattened papillae (Fig. 2B′, C′, E′, F′),

Papillae density and coverage showed strong variation in *glh6* mutant trichomes (Fig. 2F, G, J). This mutant also showed high variation in trichome branching and size (Supplementary Figs S1 and S2, and Supplementary Table S1 at *JXB* online) suggesting that papillae formation defects in the *glh6* mutant trichomes may be an indirect consequence of developmental arrest at various stages.

**Fig. 3.**
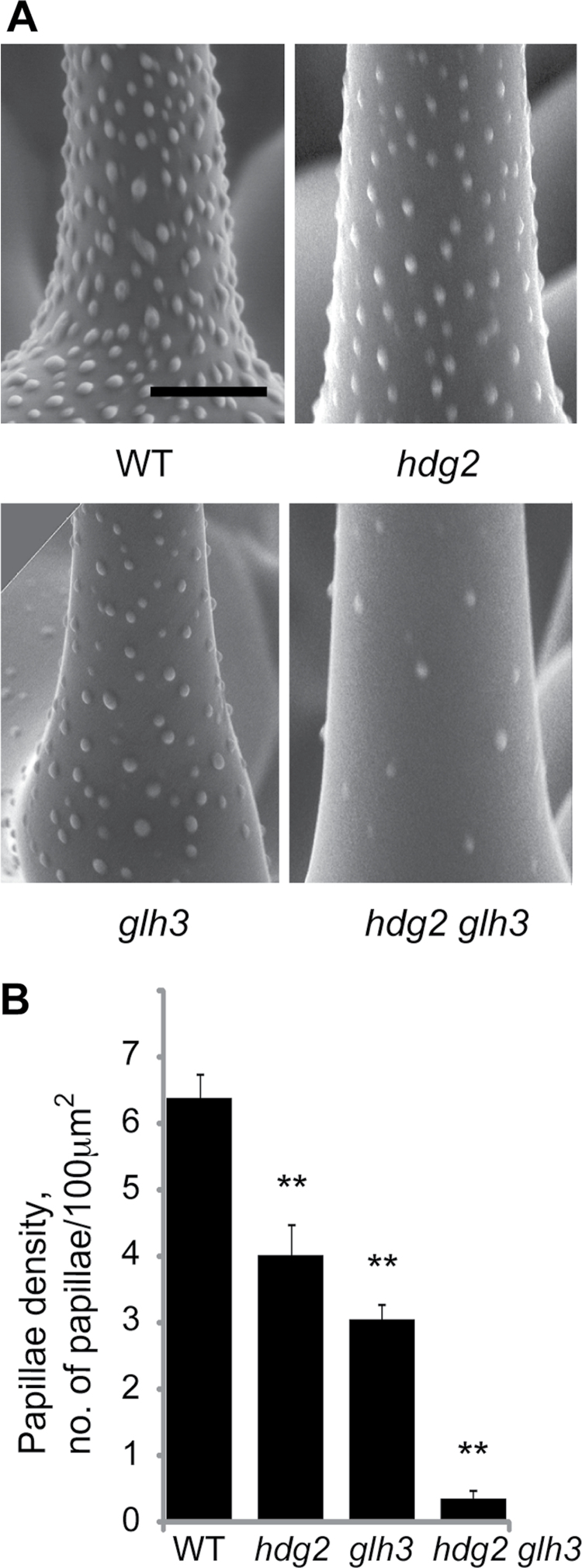
Genetic interaction between *hdg2* and *glh3* mutants. (A) Scanning electron microscopy images of the trichome surface in the wild type, *hdg2*, *glh3*, and the *hdg2 glh3* double mutant. (B) Comparison of papillae density in wild type, *hdg2*, *glh3*, and the *hdg2 glh3* double mutant. For each genotype, *n*=5. **P<0.01. Bar, 20μm. WT, wild type.

Weaker papillae defects in the *glh3* mutant resembled those of the *hdg2* glassy trichome mutant, where papillae develop at a low density (Marks *et al.*, 2009). To test the genetic relationships between these two weak glassy mutants, we created double mutants and inspected their trichome papillae. Compared with single mutants, trichomes of *hdg2 glh3* double mutants had more-severe papillae defects, forming fewer papillae (Fig. 3). This additive double-mutant phenotype suggested that the *HDG2* and *GLH3* genes promote papillae formation independently and that the *GLH3* gene is not a probable target of the *HDG2* transcription factor.

Taken together, our data suggested that *GLH* genes promote papillae development and that papillae confer light-reflective properties on trichomes.

### Trichomes of *glh2* and *glh4* mutants deposit less cellulose

The glassy trichome mutants *tbr* and *cpr5* have been shown to have reduced birefringence and cellulose deposition (Potikha and Delmer, 1995; Brininstool *et al.*, 2008; Bischoff *et al.*, 2010).

**Fig. 4.**
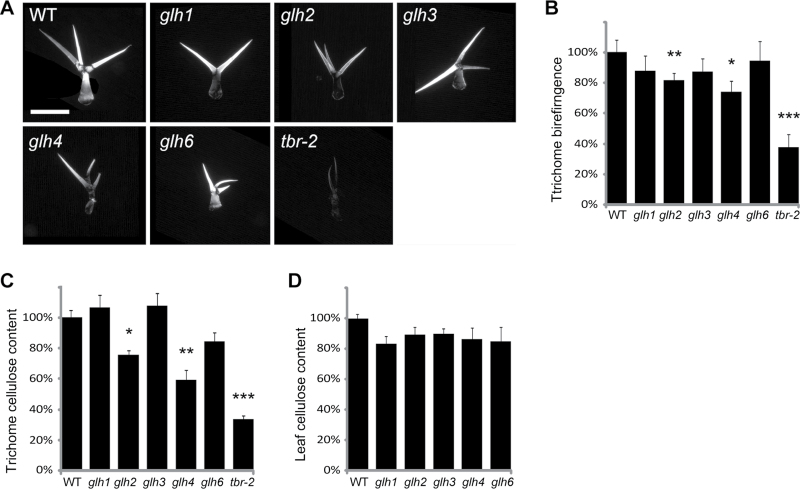
Cellulose quantification in *glh* mutants. (A) Trichome birefringence in *glh* mutants. Bar, 200 μm. (B) Quantitative analysis of trichome birefringence in *glh* mutants. The numbers of tested samples were: *n*=15 for wild type, *glh3*, *glh4*, *glh6*, and *tbr-2*; *n*=19 for *glh*; and *n*=30 for *glh2*. Significant differences relative to wild type are indicated by asterisks: *P<0.05, **P<0.01, and ***P<0.001. (C) Relative cellulose content in trichomes. The numbers of tested samples were: *n*=18 for wild type; *n*=10 for *glh1*; *n*=3 for *glh2*; *n*=5 for *glh3*; *n*=4 for *glh4*; *n*=12 for *glh6*; and *n*=6 for *tbr-2*. (D) Relative cellulose content in leaves. The number of tested samples was *n*=3 for all plants. Measured differences were small and not statistically significant. WT, wild type.

To test whether *glh* mutations affected birefringence, which is caused by highly ordered crystalline cellulose in plant cell walls, we used polarized light microscopy. Trichomes of the *tbr-2* mutant were used as a control and showed the strongest birefringence defect. Birefringence was also visibly reduced in *glh2* and *glh4* but not in *glh1*, *glh3*, and *glh6* mutants (Fig. 4A, B). These observations suggested that cellulose deposition in trichomes may be affected by *glh2* and *glh4* mutations.

To test whether *GLH* genes play a role in cellulose deposition we quantified relative cellulose amounts in the trichomes and leaves of *glh* mutants using a modified Updegraff method (Updegraff, 1969) and by normalizing the trichome cellulose amounts by adjusting to the numbers of isolated trichomes. As the *glh1*, *glh2*, *glh4*, and *glh6* mutants grew and developed slower than the wild type (Supplementary Fig. S2), leaves of the same developmental stage (rosette plants at the beginning of bolting) were used for trichome isolation. The *tbr* mutant, which was shown previously to have less cellulose in trichomes (Bischoff *et al.*, 2010), was used as a control. In our experiments, *tbr* trichomes contained 34% of the wild-type cellulose level (Fig. 4C), which is similar to previously published data. We did not detect a significant change in trichome cellulose in *glh1* and *glh3* mutants. However, *glh2* and *glh4* mutants showed a significant reduction in trichome cellulose, containing 64 and 59% of the wild-type levels, respectively (*P*<0.01 for both mutants). Trichomes of the *glh6* mutant showed a moderate reduction in cellulose to 84% of the wild-type content, but this difference was not statistically significant (*P*=0.08).

The observed cellulose reduction in *glh2*, *glh3*, and *glh6* trichome could be caused either by decreased cellulose content in trichome walls or by a decrease in trichome size. To distinguish between these two possibilities, we measured trichome length in all *glh* mutants. We found that *ghl* mutations did not affect trichome size, except for the *glh6* mutant trichomes, which showed a 31% reduction in combined length of trichome stem and branches (Supplementary Fig. S1). This suggested that the observed cellulose reduction in *glh6* trichomes could, at least partially, be due to a decrease in trichome size. Conversely, we concluded that the *GLH2* and *GLH4* genes promote cellulose deposition in trichome cell walls.

Measurements of the cellulose content in leaves did not show significant changes in *glh* mutants (Fig. 4D), suggesting that the *GLH2* and *GLH4* genes primarily affect cellulose deposition in trichomes.

### The *glh6* mutant is affected in cuticle formation

**Fig. 5.**
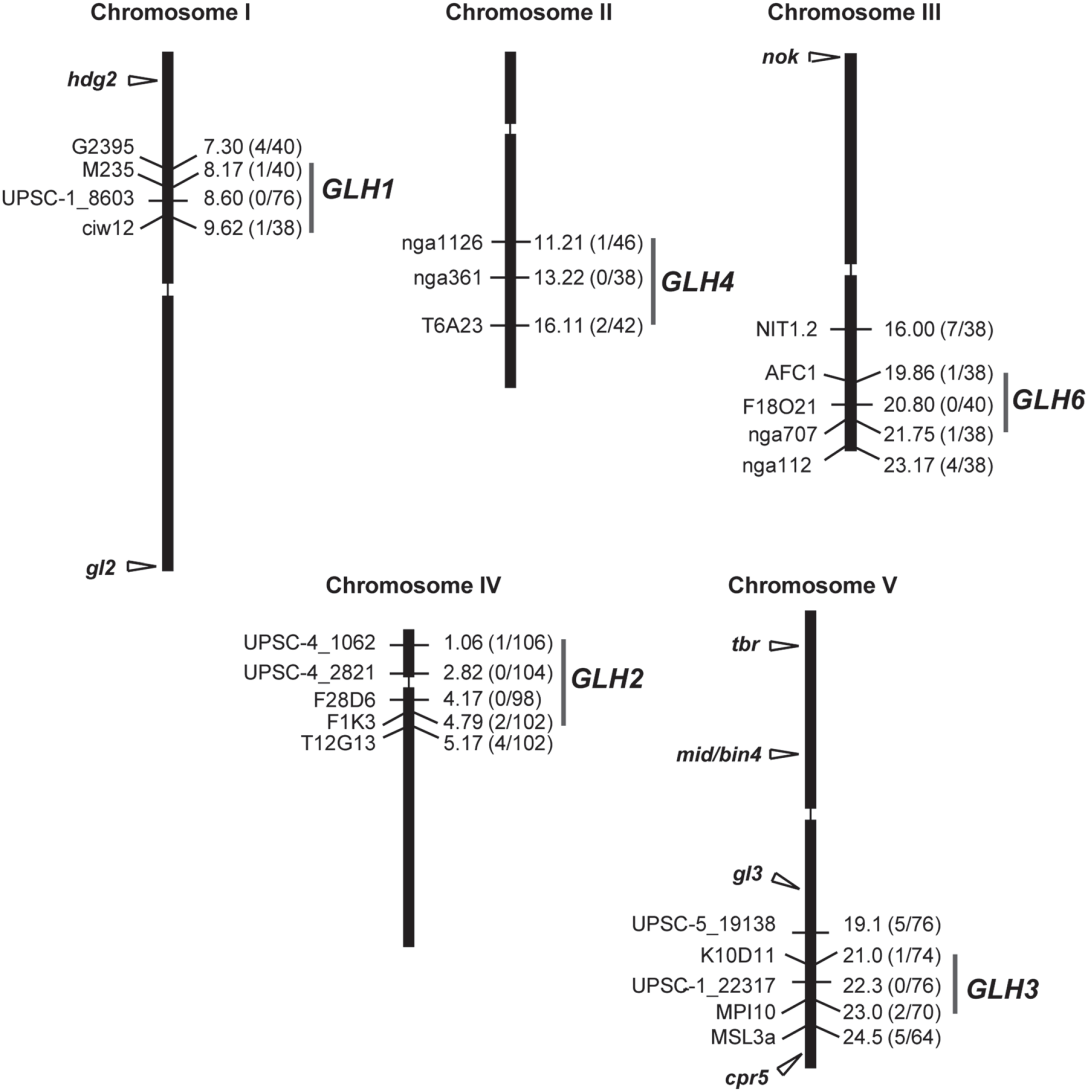
Chromosomal positions of *GLH* genes and previously characterized mutants with glassy trichomes. Mapping markers are shown to the left of the chromosome depictions, numbers to the right indicate their positions in megabase pairs. The number of detected recombination events and the total number of chromosomes tested in F2 mapping populations are shown in parentheses.

The outer covering layer of the epidermal cell wall, the cuticle, has light-reflective properties, and mutants defective in cuticle formation, such as *eceriferum*, appear bright green due to reduced light scattering by the cell surface (Koornneef *et al.*, 1989; Rashotte *et al.*, 2004). To test cuticle integrity in *glh* mutants, we stained leaves with TB dye, which binds to cell-wall polysaccharides but cannot permeate the intact cuticle layer (Tanaka *et al.*, 2004). We found that *glh6* mutant trichomes and leaves were stained with TB (Supplementary Fig. S3 at *JXB* online), suggesting a role for *GLH6* in cuticle formation. The determined chromosomal interval of the *glh6* mutation (Fig. 5) contains the *ECERIFERUM10* (*CER10*) gene, which was shown previously to be involved in cuticle formation (Zheng *et al.*, 2005). Therefore, we performed a complementation test between the *glh6* and *cer10-2* mutants. No complementation was found, suggesting that *glh6* is a new *cer10* allele.

### The *GLH1*, *GLH2*, *GLH3*, and *GLH6* genes are involved in trichome branching

In addition to glassy cell walls, trichomes in some of the *glh* mutants showed cell morphogenesis defects. On average, trichomes on *glh1*, *glh2*, and *glh6* leaves formed fewer branches. Compared with wild-type leaves with 79% three-branched and 21% four-branched trichomes, *glh1* and *glh2* mutants had 1 and 3% two-branched trichomes, respectively, 1% four-branched trichomes, and the majority (96–97%) were three-branched (Supplementary Fig. S2 and Supplementary Table S1). In contrast, *glh3* mutants formed more trichome branches exhibiting 29% four-branched and 1% five-branched trichomes.

Although on average *glh6* mutant trichomes formed fewer branches, we also observed trichomes forming with up to five branches (Supplementary Fig. S2). This suggests that the *GLH*6 gene is important for formation of trichomes with a typical number of three or four branches, having functions in both promoting branching and suppressing overbranching.

### *GLH* gene mapping

To confirm that *glh* mutants represented distinct genetic loci, we determined the chromosomal position of the *GLH* genes using PCR-based markers (Lukowitz *et al.*, 2000). In the first step, *GLH* genes were positioned on chromosomes using bulked segregant analysis. Narrow intervals were then determined for each of the five *GLH* genes (Fig. 5). Analysis of the TAIR database and *Arabidopsis* loss-of-function mutant dataset (Lloyd and Meinke, 2012) did not reveal any genes with a reported glassy mutant phenotype in the indentified intervals. This suggested that the *GLH1*, *GLH2*, *GLH3*, and *GLH4* loci are not yet functionally characterized or, as is the case for the *GHL6/CER10* gene, the trichome cell-wall phenotypes have not been reported.

## Discussion

Trichome cells deposit large amounts of cell-wall material, forming conspicuously thick cell walls (Marks *et al.*, 2008). Most of the cell-wall material is deposited during the cell-wall maturation phase (Szymanski *et al.*, 1998), after trichomes have formed branches and finished radial expansion.

In mutants, such as *gl2*, *mid*/*bin4*, and *cpr5*, trichome growth and development stops at early stages and trichomes appear glassy (Kirik *et al.*, 2007; Brininstool *et al.*, 2008; Marks *et al.*, 2009). In the *gl3-sst* mutant, where trichomes are not reduced in size but show early developmental arrest, trichomes also appear glassy and form fewer papillae on their cell walls (Esch *et al.*, 2003). Thus, the glassy trichome phenotype may be caused by developmental arrest before the trichome cell-wall maturation phase.

In this EMS-mutagenesis screen, we selected glassy trichome mutants with no strong changes in trichome size or branch number. Therefore, *GLH* genes isolated here are likely to function at the last step in trichome development and may promote the transition from the stage of diffuse growth (stage 5) to the cell-wall maturation stage (stage 6) (Szymanski *et al.*, 1998). Alternatively, rather than having developmental roles, *GLH* genes may be involved in metabolism or deposition of trichome cell-wall components.

### *GLH2*, *GLH4*, and *GLH6* genes have pleiotropic functions in trichome cell-wall maturation

Trichome cell walls in *glh2* and *glh4* mutants displayed distinct papillae defects and accumulated less cellulose: *glh2* trichomes formed almost no papillae, whereas *glh4* mutants formed smaller papillae at a normal density. Trichome length and branching were not strongly affected in these mutants, suggesting that trichome development was normal up to the cell-wall maturation stage. The *glh4* mutant phenotype was more pleiotropic and plants were strongly stunted in growth. Phenotypic differences between these mutants suggested that the *GLH2* and *GLH4* genes function in separate pathways that regulate papillae formation and trichome cellulose deposition.

Both trichome cellulose deposition and papillae formation depend on the *GLH2* function. A similar phenotype is caused by the *Arabidopsis tbr* mutation (Potikha and Delmer, 1995). Both mutants show reduced birefringence and cellulose content in trichomes (Fig. 4A–C) (Potikha and Delmer, 1995; Bischoff *et al.*, 2010). *TBR* and its homologous *TBR-like* (*TBL*) genes encode putative transmembrane proteins with a plant-specific DUF231 domain. Although the molecular function of the TBR protein is not known, it was suggested that it might support secondary cell-wall formation by promoting pectin methylesterification, as etiolated hypocotyls of *tbr* and *tbl3* mutants had increased pectin methylesterase activity and showed reduced pectin esterification (Bischoff *et al.*, 2010). Pectins are present in large amounts in trichome cell walls (Marks *et al.*, 2008) and may play a role in cellulose deposition, as suggested by their binding to cellulose (Zykwinska *et al.*, 2005). Thus, it is conceivable that reduced cellulose content in *tbr* mutants may be a secondary consequence of deficiency in non-cellulosic cell-wall polysaccharides. Previous data indicate complex relationships between components of the cell wall, so that changes in deposition, structure, or modification of one cell-wall component often affected others. For example, it has been shown that the *IXR8*/*GAUT12* gene, which regulates production of glucoronoxylan and homogalacturonan cell-wall polysaccharides, is important for anisotropic cell expansion and cellulose content (Persson *et al.*, 2007). Conversely, cellulose deposition can influence pectin formation, as several mutants deficient in cellulose production exhibited increased pectin contents (His *et al.*, 2001). No members of the TBR/TLB family are present in the *GLH2* mapping interval on chromosome 4, and therefore it is unlikely that the *GLH2* gene belongs to this family. Identification of the *GLH2* gene together with detailed phenotypic analysis of the mutant cell wall will address the relationship between the *GLH2* and *TBR* genes and will help to elucidate molecular mechanisms regulating both papillae and cellulose deposition during trichome cell-wall maturation.

Trichomes on *glh6* mutants had fewer papillae and were variable in size, branch number, and branch shape, often forming rounded branch tips, indicating arrest at stages 3, 4, 5, and 6 of trichome development (Szymanski *et al.*, 1998). This variation in phenotype was observed even within one leaf, with less-developed trichomes showing a stronger glassy phenotype. Mapping the *glh6* mutation placed it into the 1.9 Mbp interval on the lower arm of chromosome 3. This interval contains the *CER10* gene, which shows a similar loss-of-function phenotype with the *glh6* mutant. Both mutant plants have stunted growth, produce sterile flowers, deposit less cuticular wax, and occasionally have fused trichomes when grown in high humidity (Supplementary Fig. S3B; Zheng *et al.*, 2005). Our complementation tests revealed that *glh6* is a new *cer10* allele. Although the glassy trichome phenotype was not described for the *cer10* mutant, the *glh6* glassy phenotype is relatively weak and not uniform among the trichomes. Observed variability of *glh6* mutant trichomes may explain why the glassy phenotype has not been reported for the *cer10* mutants. The *CER10* gene encodes the enoyl-CoA reductase, which is required for very-long-chain fatty acid synthesis (Zheng *et al.*, 2005). It has been suggested that altered composition of very-long-chain fatty acid sphingolipids in *cer10* mutants causes pleiotropic developmental changes.

### Trichome papillae formation and function

Targeted cell-wall deposition events leading to formation of trichome papillae, molecular composition, and functions of papillae are not understood. To uncover the molecular mechanisms of cell-wall maturation in trichomes, genes involved in papillae formation need to be identified and characterized.

Trichomes of the *glh1* mutant showed wild-type levels of cellulose and normal birefringence. At the same time, the *glh1* trichomes had lost most of the surface papillae. This suggests that papillae development and formation of crystalline cellulose are independent of each other. However, these two processes are likely to share common upstream pathways that involve the *TBR* and *GLH2* genes, as *tbr* and *glh2* papillae-less mutants also have reduced cellulose levels in trichomes.

Hemicelluloses have been linked to papillae formation in several studies. Double mutants in the functionally redundant *XXT1* and *XXT2* genes, encoding xylosyltransferases, displayed collapsed papillae on their trichomes (Briggs *et al.*, 2006; Cavalier *et al.*, 2008). The papillae also appear wrinkled and collapsed on *mur2* and *mur3* trichomes (Vanzin *et al.*, 2002; Madson, 2003). The *MUR2* gene accounts for all of the xyloglucan fucosyltransferase activity in *Arabidopsis*, while MUR3 acts as a galactosyltransferase targeting the third xylose residue in the XXXG xyloglucan core structure. Scanning electron microscopy images of the trichome surface have shown that the papillae phenotype is less pronounced in *mur2* and *mur3* mutants than in *glh* mutants (Madson, 2003). Thus, the relatively weak papillae phenotype in the hemicellulose mutants suggests that hemicelluloses provide a minor contribution to trichome papillae formation.

Trichomes on *glh1* and *glh3* mutants displayed strong papillae defects, affecting both density and coverage of the trichome surface (Fig. 2). These mutants did not show any alterations in cellulose and cuticular wax. This suggests papillae-specific functions for the *GLH1* and *GLH3* genes during trichome cell-wall maturation. These genes may be required for synthesis or deposition of papillae components. Identification of *GLH1* and *GLH3* genes may uncover molecular mechanisms of trichome papillae formation, composition, and function.

### Conclusions

Just as roughening of clear glass turns it into frosted glass by introducing surface irregularities, trichome papillae introduce surface non-uniformities that can scatter light. Indeed, all *glh* mutants displayed trichome defects in papillae, forming fewer or less-developed papillae. This suggests that the trichome glassy phenotype is caused by reduced light reflection due to diminished scattering on a smooth trichome surface of papillae-less *glh* trichomes. As a result, more light can pass through the *glh* trichomes (Fig. 1C).

Our data indicated that papillae increase light reflection of trichomes. This optical property may play an important role in previously suggested trichome functions in leaf temperature regulation (Dell and McComb, 1979; Klich, 2000), in protection against UV-B radiation (Manetas, 2003), and in protection of the photosystem II in young leaves against excessive light (Karabourniotis *et al.*, 1992; Karabourniotis *et al.*, 1993). The *glh* mutants provide a tool to address physiological functions of trichome papillae in future studies. Identification of the *GLH* genes will provide an insight into mechanisms of localized deposition of materials on the outer cell-wall surface and will expand our understanding the cellulose deposition mechanisms in trichome cells.

## Supplementary data

Supplementary data are available at *JXB* online.

Supplementary Table S1. Trichome branching in *glh* mutants.

Supplementary Fig. S1. Relative trichome length of *glh* mutants compared to wild type.

Supplementary Fig. S2. Effects of *glh* mutations on plant development.

Supplementary Fig. S3. *glh6* mutants have cuticle defects.

Supplementary Data

## Abbreviations:

CAPS: cleaved amplified polymorphic sequences
EMS: ethyl methanesulfonate
MS: Murashige and Skoog
TB: toluidine blue.
