# Supplementary material for: Arabidopsis GLASSY HAIR genes promote trichome papillae development
Source: J Exp Bot. 2013 Sep 7;64(16):4981–91. doi: 10.1093/jxb/ert287 (PMC3830481; doi:10.1093/jxb/ert287)
Supplement: Supplementary Data [file supp_64_16_4981__index.html]

 Arabidopsis GLASSY HAIR genes promote trichome papillae development — Arabidopsis GLASSY HAIR genes promote trichome papillae development — Supplementary Data 

# *Arabidopsis GLASSY HAIR* genes promote trichome papillae development

## Supplementary Data

Data files

**Files in this Data Supplement:**

- Supplementary Data - Supplementary Data
