# Supplementary material for: Arabidopsis GLASSY HAIR genes promote trichome papillae development
Source: J Exp Bot. 2013 Sep 7;64(16):4981–91. doi: 10.1093/jxb/ert287 (PMC3830481; doi:10.1093/jxb/ert287)
Supplement: Supplementary Data [file supp_ert287_jexbot102301_file001.pdf]

Supplementary material

Arabidopsis GLASSY HAIR genes promote trichome papillae development

Bangxia Suo, Stephanie Seifert and Viktor Kirik

Table S1. Trichome branching in *glh* mutants.

Trichome tips	**WT**	***glh1***	***glh2***	***glh3***	***glh4***	***glh6***
1	0	0	0	0	0	52
2	0	12	4	0	0	85
3	246	338	265	276	241	44
4	65	1	3	117	36	3
5	0	0	0	4	0	2
Total	311	351	272	397	277	186

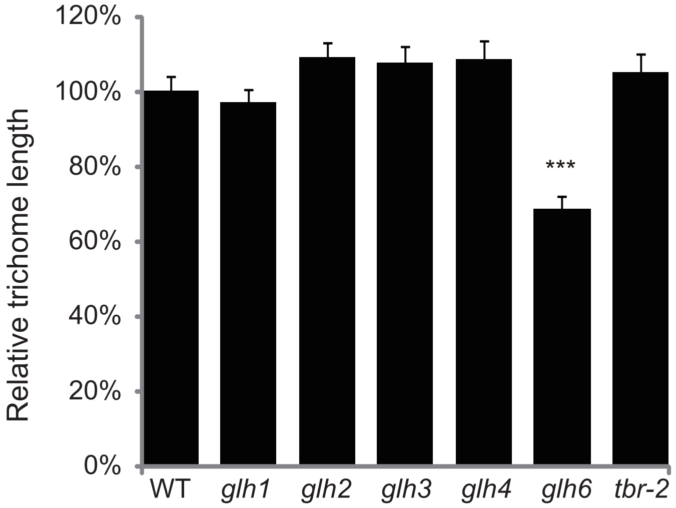

Figure S1. Relative trichome length of *glh* mutants compared to wild type. Trichomes length was calculated as a sum of stem and branch lengths. 40 trichomes isolated from fully expanded young leaves were measured for each genotype. *glh6* trichomes were significantly smaller than wild type, ***$p < 0.001$.

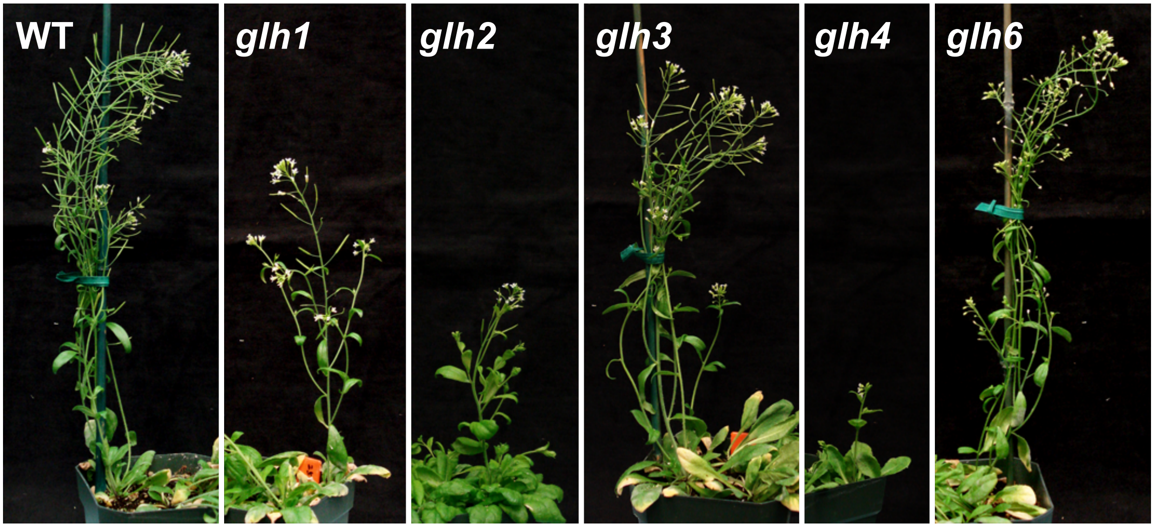

Figure S2. Effects of *glh* mutations on plant development. 6-week-old plants are shown. Note stunted growth of *glh1*, *glh2* and *glh4* mutants.

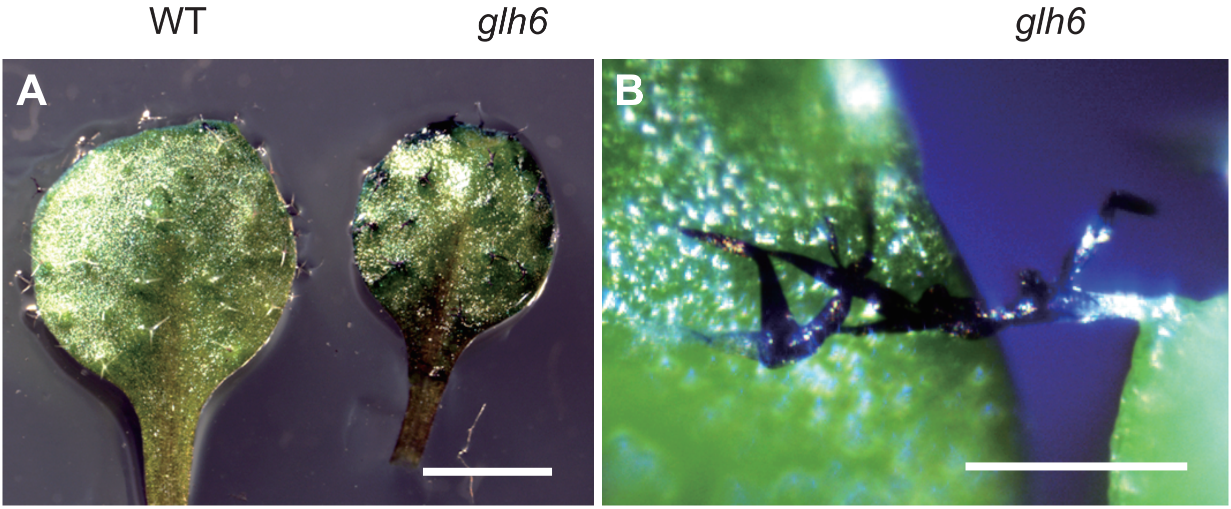

Figure S3. *glh6* mutants have cuticle defects. Permeability of wild type, *glh1*, *glh2*, *glh3*, *glh4* and *glh6* leaves were examined by the TB test. (A) Trichomes on *glh6* leaves showed strong staining with TB. Negative TB staining of other *glh* mutants is not shown. (B) Higher magnification shows occasionally fused TB-positive trichomes. Scale bar =2 mm for A and =200 μm for B.
